# Transferability of bone phenotyping and fracture risk assessment by μFRAC from first-generation high-resolution peripheral quantitative computed tomography to second-generation scan data

**DOI:** 10.1093/jbmr/zjae039

**Published:** 2024-03-04

**Authors:** Annabel R Bugbird, Danielle E Whittier, Steven K Boyd

**Affiliations:** McCaig Institute for Bone and Joint Health, Cumming School of Medicine, University of Calgary, Calgary, AB T2N 4Z6, Canada; McCaig Institute for Bone and Joint Health, Cumming School of Medicine, University of Calgary, Calgary, AB T2N 4Z6, Canada; Department of Cell Biology and Anatomy, Cumming School of Medicine, Alberta Children’s Hospital Research Institute, University of Calgary, Calgary, AB T2N 4N1, Canada; McCaig Institute for Bone and Joint Health, Cumming School of Medicine, University of Calgary, Calgary, AB T2N 4Z6, Canada

**Keywords:** high-resolution peripheral quantitative computed tomography, bone microarchitecture, cross-calibration, osteoporotic fracture, phenotyping, fracture risk

## Abstract

**Introduction:**

The continued development of high-resolution peripheral quantitative computed tomography (HR-pQCT) has led to a second-generation scanner with higher resolution and longer scan region. However, large multicenter prospective cohorts were collected with first-generation HR-pQCT and have been used to develop bone phenotyping and fracture risk prediction (μFRAC) models. This study establishes whether there is sufficient universality of these first-generation trained models for use with second-generation scan data.

**Methods:**

HR-pQCT data were collected for a cohort of 60 individuals, who had been scanned on both first- and second-generation scanners on the same day to establish the universality of the HR-pQCT models. These data were each used as input to first-generation trained bone microarchitecture models for bone phenotyping and fracture risk prediction, and their outputs were compared for each study participant. Reproducibility of the models were assessed using same-day repeat scans obtained from first-generation (*n* = 37) and second-generation (*n* = 74) scanners.

**Results:**

Across scanner generations, the bone phenotyping model performed with an accuracy of 93.1%. Similarly, the 5-year fracture risk assessment by μFRAC was well correlated with a Pearson’s (*r*) correlation coefficient of *r* > 0.83 for the three variations of μFRAC (varying inclusion of clinical risk factors, finite element analysis, and dual X-ray absorptiometry). The first-generation reproducibility cohort performed with an accuracy for categorical assignment of 100% (bone phenotyping) and a correlation coefficient of 0.99 (μFRAC), whereas the second-generation reproducibility cohort performed with an accuracy of 96.4% (bone phenotyping) and a correlation coefficient of 0.99 (μFRAC).

**Conclusion:**

We demonstrated that bone microarchitecture models trained using first-generation scan data generalize well to second-generation scans, performing with a high level of accuracy and reproducibility. Less than 4% of individuals’ estimated fracture risk led to a change in treatment threshold, and in general, these dissimilar outcomes using second-generation data tended to be more conservative.

## Lay Summary

Establishing the universality of first-generation-trained HR-pQCT prediction models on second-generation scan data is important to move the bone microarchitecture field forward. We found that despite the difference in resolutions between the two HR-pQCT generations, models developed with first-generation data generalized well to second-generation systems. This avoids unnecessarily repeating complex studies.

## Introduction

Osteoporosis is a degenerative disease, characterized by increased bone fragility and susceptibility to fracture [1]. Currently, osteoporosis is attributed to over 9 million fractures worldwide a year [2], playing a major role in morbidity and mortality among the aging population [3, 4]. Despite the significant impact on health and quality of life, osteoporosis typically remains undiagnosed until a fragility fracture takes place [5].

The current gold standard for osteoporotic fracture prediction and diagnosis is based on areal bone mineral density (aBMD) captured using dual X-ray absorptiometry (DXA). Fracture risk assessment tools such as FRAX, CAROC, and Garvan can incorporate DXA aBMD to predict the probability of osteoporotic fracture, but there are limitations resulting in a low sensitivity [6–10]. The majority of people (>50%) who experience a fragility fracture are not radiologically classified as osteoporotic and do not meet the management criteria as defined by the World Health Organization (T-score < −2.5) [11]. This in part is due to the fact the DXA does not provide information on bone structure, a major contributor to bone strength [12].

With the advent of three-dimensional (3D) high-resolution peripheral quantitative computed tomography (HR-pQCT), it is possible to capture microarchitectural, geometric, and density in one measurement [11]. Since its introduction in the early 2000s, the most recent version is a so-called second-generation HR-pQCT device that features a higher resolution and longer scan region [13]. Recent studies have demonstrated that bone parameters measured using HR-pQCT imaging provide improved prediction of fracture [14, 15]. However, the numerous and complex parameters provided by HR-pQCT can be challenging to interpret. Recently, machine learning techniques have been developed to overcome this challenge [16, 17]. These models from our lab include (1) a bone phenotyping model, which identifies common combinations of bone properties, known as phenotypes, that are associated with varying levels of fracture risk [16], and (2) the microarchitecture fracture risk assessment calculator (μFRAC) model, which uses HR-pQCT bone parameters and other relevant clinical information to output a 5-year probability of osteoporotic fracture [17].

A major challenge is that these models were trained using first-generation HR-pQCT imaging with contributions from research groups worldwide who collected prospective data. With the advent of second-generation HR-pQCT, it is not known how well these models will perform given its improved imaging capabilities. However, it is challenging to re-develop these models as it would require establishing new large multicentre prospective cohorts using the newer HR-pQCT with barriers of time and cost.

The aim of this study is to determine the performance of the bone phenotyping and μFRAC models on second-generation scan data, to understand the generalizability of these bone microarchitecture-focused HR-pQCT models. Additionally, we calculate the reproducibility of these two models for both first- and second-generation data using repeat same-day scans.

## Materials and methods

### Participants

For establishing the generalizability of HR-pQCT models across-scanner generations, a total of 60 participants (42 female and 18 male) aged between 55 and 71 years old were recruited from Calgary, Alberta, and the surrounding area. These participants were scanned on both first-generation (XT1) and second-generation (XT2) scanners on the same day. This cohort has previously been used for cross-calibration of standard HR-pQCT measurements [18]. All participants had a femoral neck T-score > −2.5, were not taking bone active medication, and any chronic medical conditions were stable for at least 2 years prior to study enrollment [19]. All participants provided written and informed consent before involvement in the study, which was approved by the Conjoint Health Research Ethics Board at the University of Calgary.

### Medical imaging

The standard in vivo scanning protocols for XtremeCT (82.0 μm, Scanco Medical AG, Brütisellen, Switzerland) and XtremeCT II (60.7 μm, Scanco Medical AG, Brütisellen, Switzerland) were used [13]. However, as these scans were done upon receiving the XtremeCT II system in 2014, 6 years prior to the establishment of the guidelines, our offset was 0.5 mm more proximal than the guidelines recommend. Participants were scanned at their non-dominant radius and left tibia for all scans. If there was a prior fracture, surgery, or implant at the scan site, the opposite limb was scanned. Scans were acquired at a fixed offset of 9.5 and 22.5 mm proximal to the reference line for the radius and tibia for both scanners. The XT1 scans captured 110 slices (9.0 mm) and the XT2 scans captured 168 slices (10.2 mm). All scans were graded for motion artifacts on a scale of 1 (no motion) to 5 (significant streaking and discontinuities in the cortex), with all scores of 4 or higher being excluded from further analysis.

Femoral neck areal BMD (aBMD) was measured using DXA (GE Lunar iDXA; GE Healthcare, Piscataway, NJ; encore v16). The left femoral neck was scanned for all participants, except in the case of participants with a prior left hip fracture, where the contra-lateral side was scanned.

Individuals in the cohort who did not have both a radius and tibia HR-pQCT scan for XT1 and XT2, as well as a DXA scan, were excluded from further analysis.

### HR-pQCT analysis

All analyses were performed on the full image data from each of the scanners, despite the difference in scan region length, to be representative of the standard outputs acquired from each scanner. The analysis was performed following the manufacturer’s standard patient protocol for each scanner [13]. The periosteal and endosteal contours were identified semiautomatically and manually corrected when needed [20]. The bone parameters acquired following analysis included: total bone mineral density (TtBMD), cortical BMD (CtBMD), trabecular BMD (TbBMD), total area (TtAr), cortical area (CtAr), trabecular area (TbAr), trabecular number (TbN), trabecular inhomogeneity (SdTbN), and cortical thickness (CtTh) at both the radius and tibia. XT1 measured CtTh was derived by dividing the volume of the cortical compartment by the periosteal surface; in XT2, CtTh was measured as the mean spacing of the periosteal and endocortical surfaces using the distance transformation method.

Estimated failure load was calculated using linear micro-finite element (μFE) modeling on the segmented HR-pQCT images for both scanners (FAIM, v8.0; Numerics88 Solutions Ltd, Calgary, Canada). A tissue modulus of 6829 GPa for XT1 and 8748 GPa for XT2 and a Poisson ratio of 0.3 were applied to the bone. A linear axial compression test with 1% compressive strain was applied to the bone, and failure was defined as a yield criterion of 2% critical volume and 0.7% critical strain. A convergence criterion of 1 × 10^-6^ was selected.

### Bone phenotyping model

The bone phenotyping model developed in our lab [16] was trained using the fuzzy c-means algorithm and identifies three bone phenotypes described as *low density*, *healthy*, and *low volume*. The outputs of the model include a membership coefficient to each of the phenotypes (i.e. the probability the individual belongs to each of the three phenotypes) and a categorical phenotype classification (i.e. the phenotype with the highest membership coefficient). The bone phenotypes are associated with fracture risk, with individuals classed as a *low density* phenotype having the highest fracture risk, and individuals classified as *healthy* phenotype having the lowest fracture risk. The bone phenotyping model was trained using a large population cohort (*n* = 6836) obtained from the Bone Microarchitecture International Consortium (BoMIC) consisting of both male and female participants with prospective fracture information. Individuals were scanned using first-generation HR-pQCT with a fixed offset of 9.5 and 22.5 mm at the radius and tibia, respectively.

### μFRAC model

The μFRAC model was developed in our lab using random survival forests and the primary output is the 5-year risk of a major osteoporotic fracture (MOF) [17]. There are three variations of the μFRAC model, including simplifications that do not include FN aBMD and all clinical risk factors except for age (μFRAC') and another further simplified by removing μFE-estimated failure load at the radius and the tibia (μFRAC"). The μFRAC model was trained using the BoMIC cohort described above.

### Assessing the universality of the bone phenotyping and μFRAC models

To determine the universality of the models to be used for either XT1 or XT2 input data (across scanner generations), a number of statistical tests were performed. The agreeability of phenotyping was based on the phenotype membership (a three-component unit vector) between scanners using the Pearson’s (*r*) correlation coefficient of the cluster membership obtained for XT1 and XT2 data. Additionally, phenotype agreeability was assessed by the cosine similarity metric which measures the similarity between the membership values between the XT1 and XT2 data, and Euclidean distance which measures the distance between the two points in space. To visually assess the differences between the membership coefficients between scanner generations, Bland–Altman plots were generated for each of the three clusters. Categorical phenotype classification was assessed using a confusion matrix, as well as estimations of accuracy and individual cluster sensitivity. Fracture risk was assessed by calculating the agreeability of the 5-year MOF risk between the two scans using the Pearson’s (*r*) correlation coefficient for each of the three μFRAC models that included varying information (eg with or without DXA or μFE). To visually assess the differences between the predicted fracture risk outcomes between scanner generations, Bland–Altman plots were generated for each of the μFRAC model variants.

### Reproducibility

Within-scanner generation reproducibility was performed for both the bone phenotyping and μFRAC models, to provide a reference for the comparison between XT1 and XT2 data. Participants were selected from an unrelated XT1 precision cohort (*n* = 37), who had undergone repeat HR-pQCT scanning of the distal radius and distal tibia on the same day [21]. The same scanning protocol and analysis for XT1 data were followed as outlined above. To assess the reproducibility of the models on the cohort, the bone phenotype membership coefficients were compared using Pearson’s correlation coefficient, the cosine similarity metric, and Euclidean distance between the two data points. The categorical phenotype classification was assessed using accuracy and sensitivity. To determine the reproducibility of the μFRAC models on the reproducibility cohort compared with the between-scanner reproducibility, the Pearson’s correlation coefficient was used to compare the 5-year MOF risk between the two data points. Similarly, to determine the reproducibility of both models on XT2 data, a cohort was selected from an unrelated XT2 precision cohort (*n* = 74) [18]. The same scanning protocol and analysis for XT2 data were followed as outlined above, and the same statistical methods were used as described with the XT1 precision cohort.

Individuals in the XT1 and XT2 reproducibility cohorts were excluded from further analysis if, they did not have both a radius and tibia scan; either the radius or tibia scan had a motion score of >4, or they did not have a DXA scan.

### Model precision

The precision of both the bone phenotyping and μFRAC models was assessed using the root-mean-square standard deviation (RMSSD) to determine the average difference between the model outcomes, and least-significant change (LSC) calculated as 2.77 times the precision error (RMSSD) to determine the least amount of change that can be considered statistically significant [22, 23].

## Results

### Demographics for participants

For universality between scanner generations, a total of 58 participants (71% female) with a mean age of 63.4 ± 4.0 years were included in this study. The average bone parameters measured on each scanner and demographic information for the cohort are shown in Table S1. One individual experienced a previous hip fracture, and two individuals were excluded from the analysis due to a motion score of >4 at either the radius or the tibia. The reproducibility cohort used for XT1 within-scanner reproducibility consisted of 35 individuals (74% female) with a mean age of 58.2 ± 8.8 years. Two individuals were removed from the analysis due to motion scores >4 at either the radius or tibia. The reproducibility cohort used for XT2 within-scanner reproducibility consisted of 55 (47% female) with a mean age of 66.2 ± 4.9 years; 19 individuals were excluded as the results of a motion score > 4 at either the radius or tibia site.

### Bone phenotyping

**Figure 1:**
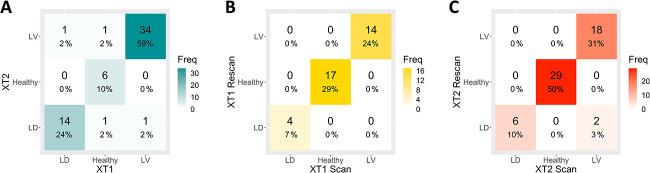
Bone phenotyping model categorical phenotype classification for (A) the XT1 and XT2 data set (across scanner), (B) the XT1 reproducibility cohort (within scanner), and (C) the XT2 reproducibility cohort (within scanner), where LD is the low density phenotypye and LV is the low volume phenotype.

**Figure 2:**
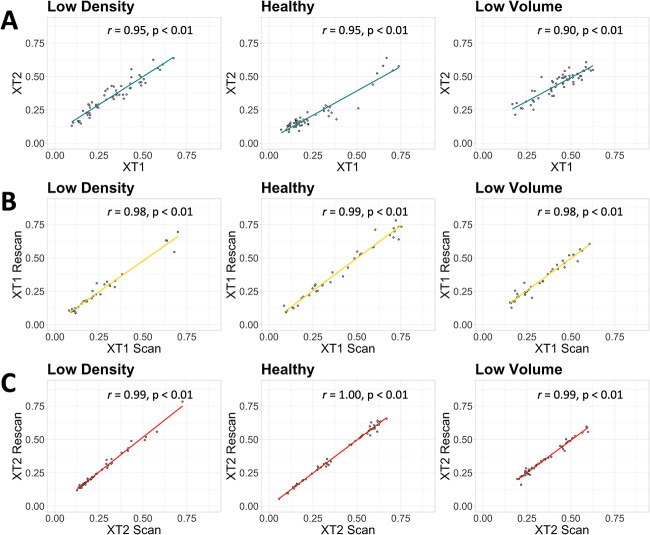
Correlation of the low density, healthy, and low volume phenotype membership values for (A) the XT1 and XT2 dataset (across scanner), (B) the XT1 reproducibility cohort (within scanner), and (C) the XT2 reproducibility cohort (within scanner).

**Figure 3:**
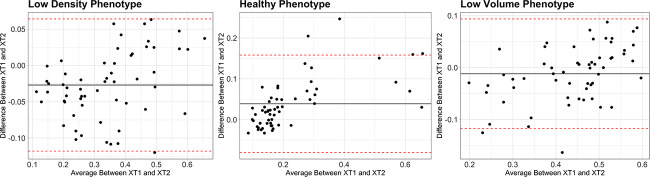
Bland–Altman plots between the membership values obtained using XT1 and XT2 data (across scanner). The plot displays the mean difference and 95% limits of agreement.

Using the XT1 scan data as the ground truth (because the original models were developed with XT1 data), 15 (25.9%) individuals from the data set were classified as *low density*, 8 (13.8 %) were classified as *healthy*, and 35 (60.3 %) were classified as *low volume* using the bone phenotyping model. A confusion matrix of the categorically assigned phenotype classification for each individual using XT2 data compared with XT1 data is shown (Figure 1A). The model performed with an accuracy of 93.1 %, with a group sensitivity of 93.3%, 75.0%, and 97.1% for the *low density*, *healthy*, and *low volume* phenotypes, respectively. An average cosine similarity of 0.99 ± 0.02 (min 0.88, max 0.99) and a Euclidean distance of 0.09 ± 0.06 (min 0.01, max 0.31) were observed between XT1 and XT2 data, which shows a high level of similarity between the predicted membership on an individual level. Additionally, a strong correlation (*r* > 0.90) between scanners was observed for all three phenotypes (Figure 2A), indicating stable classification between the scan generations. On average, differences between the membership values obtained between scanner generations tended around 0 (Figure 3) and showed no clear error bias across the phenotypes. However, there was a small positive systematic bias, where the magnitude of the differences was greater with higher membership values.

Characterizing the XT1 reproducibility cohort, 4 (11.4%) individuals were classified as *low density*, 17 (48.6%) were classified as *healthy*, and 14 (40.0%) were classified as *low volume*. The confusion matrix of the categorical phenotype classification between the two scans on XT1 was generated (Figure 1B). The predicted categorical phenotype classification between time points agreed with 100% accuracy and group sensitivity of 100% for the *low density*, *healthy*, and *low volume* phenotypes. The average cosine similarity of the XT1 reproducibility cohort was 0.99 ± 0.00 (min 0.98, max 0.99) and the average Euclidean distance 0.04 ± 0.04 (min 0.00, max 0.17). The correlation between the predicted membership values for each of the phenotypes in the validation cohort is shown in Figure 2B, and a strong correlation (*r* > 0.98) was seen in the data.

Similarly, for the XT2 reproducibility cohort, 6 (10.9%) individuals were classified as *low density*, 29 (52.7%) were classified as *healthy*, and 20 (36.4%) were classified as *low volume* (Figure 1C). The predicted categorical phenotype classification between the two time points in the XT2 reproducibility cohort was 96.4%, and group sensitivity of 100% for the *low density*, 100% for *healthy*, and 90.0% for the *low volume* phenotypes. The average cosine similarity of the XT2 reproducibility cohort was 0.99 ± 0.00 (min 0.99, max 0.99) and the average Euclidean distance 0.02 ± 0.02 (min 0.00, max 0.08). The correlation between the predicted membership values for each of the phenotypes in the validation cohort (Figure 2C) was strong (*r* > 0.99).

### μFRAC

**Figure 4:**
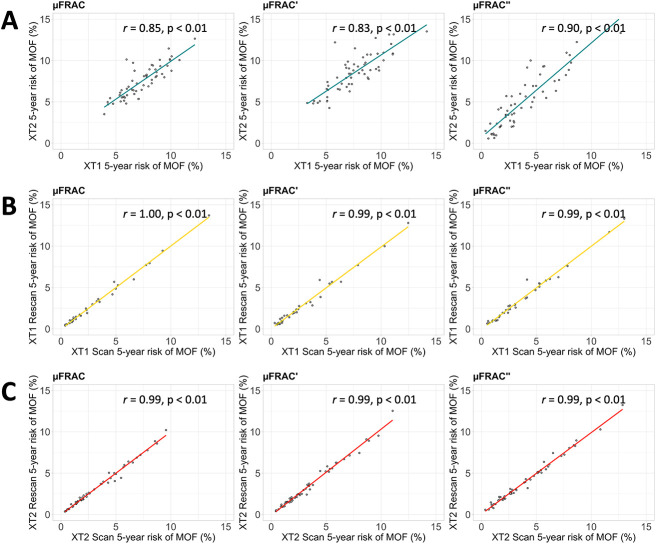
Correlation of major osteoporotic fracture (MOF) risk predicted from the μFRAC, μFRAC', and μFRAC" models for (A) the XT1 and XT2 dataset (across scanner), (B) the XT1 reproducibility cohort (within scanner), and (C) the XT2 reproducibility cohort (within scanner).

**Figure 5:**
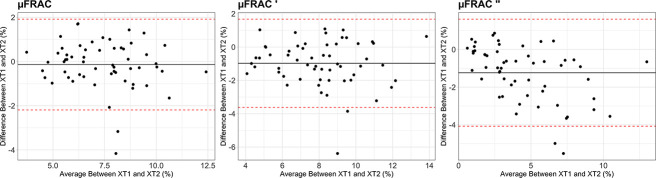
Bland–Altman plots between the μFRAC risk scores obtained using XT1 and XT2 data (across scanner). The plot displays the mean difference and the 95% limits of agreement.

The correlations between risk scores for the XT1 and XT2 scanners (between scanners) were *r* = 0.85, 0.83, and 0.90 for the μFRAC, μFRAC', and μFRAC" models, respectively, as shown in linear regressions (Figure 4A). On average, differences between the fracture risk estimates obtained between scanner generations tended around 0 (Figure 5) and showed no clear error bias across the model variations. However, a slight negative systematic bias was observed, in particular in the μFRAC" model, where the magnitude of the differences was greater with lower risk estimates. The correlation between the two scans in the XT1 reproducibility cohort (within scanner) was *r* = 1.00, 0.99, and 0.99 for the μFRAC, μFRAC', and μFRAC" models, respectively, as shown in linear regressions (Figure 4B), and *r* = 0.99 for each of the models in the XT2 reproducibility cohort (within scanner) (Figure 4C).

### Model precision

Within-scanner precision results are shown in Table 1 for both the bone phenotyping and μFRAC models. Precision (RMSSD and LSC) was similar for XT1 and XT2 data.

**Table 1:** Precision for model outcomes on the XT1 and XT2 reproducibility datasets based on RMSSD and LSC, where phenotype is represented as a membership coefficient, and μFRAC is represented as a percent 5-year fracture risk.

		**XT1 Reproducibility**		**XT2 Reproducibility**	
		RMSSD	LSC	RMSSD	LSC
	Low Density	0.03	0.09	0.15	0.40
Phenotype	Healthy	0.04	0.10	0.21	0.58
	Low Volume	0.03	0.08	0.13	0.36
	μFRAC	0.25%	0.69%	0.26%	0.73%
	μFRAC'	0.39%	1.08%	0.34%	0.93%
	μFRAC"	0.40%	1.12%	0.31%	0.86%

Abbreviations: RMSSD, root-mean-square standard deviation; LSC, least-significant change.

## Discussion

This study has established the generalizability of XT1 trained bone microarchitecture models on XT2 data sets, as well as determining the reproducibility of outputs obtained from these models for both XT1 and XT2 data. It is clear that there are differences between the morphological outcomes assessed by XT1 and XT2 [18, 24], but we determined that despite these differences, the predicted outputs between the two scan generations show a high level of agreeability. The phenotype membership correlated strongly between the scan generations, and categorical phenotyping performed with a high level of accuracy. The μFRAC model showed strong correlation between the XT1 and XT2 data, with minimal differences amounting to changes in risk category. Overall, both the bone phenotyping and μFRAC outcomes were highly reproducible with both the XT1 and XT2 data.

The bone phenotyping model was robust to the data input. The model performed with a high level of accuracy, and good group sensitivity between the XT2 data compared with XT1, in particular in the non-healthy phenotypes. However, the categorical classification of the healthy phenotype had the lowest sensitivity between scanner generations, which in part could be due to the size of the group, with only 8 individuals being classed as healthy by XT1 from the cross-calibration cohort data. Despite this, the low sensitivity in the healthy phenotype indicates that the majority of misclassifications when generalizing the model between scanner generations tended away from the healthy group, which results in a slightly more conservative estimate of the bone phenotype. This is illustrated in Figure 1, where none of the misclassifications in the confusion matrix are being classed as healthy. In addition, of the 4 misclassifications in the confusion matrix, 3 of the individuals equally belonged to two phenotypes, meaning that they could have been categorically classed as either phenotype category. This is shown in Figure 2, where there are few outliers, indicating that the membership values predicted for each of the phenotypes on XT2 were consistent with the XT1 data, and subtle changes to the phenotype membership coefficients are driving the change in categorical phenotype. Reporting membership values in addition to categorical phenotypes provides an indication of the membership strength with each phenotype; this allows individuals that share characteristics from more than one phenotype to be shown from the model output.

μFRAC was consistent for all three μFRAC model variants, and any difference of note generally resulted in a more conservative estimate. The correlation coefficients for the μFRAC, μFRAC', and μFRAC" models on the XT2 data compared with the XT1 data were slightly lower than the reproducibility cohorts (Figure 4); however, the difference between the predicted risk value for the XT1 and XT2 data was minimal (average < 1%). Although not yet validated on the μFRAC model, a useful exercise could be to evaluate our results in the context of the three risk categories defined by the 2010 clinical practice guidelines [25]: individuals who have less than a 10% of experiencing an MOF over 10 years are considered as low risk, individuals with a fracture risk of 10–20% are considered at moderate risk, and those >20% are considered at high risk. Those who are categorized as being at high risk may be recommended for pharmacologic therapy, whereas those who fall into the moderate category might be considered for pharmacologic therapy in conjunction with other relevant risk factors. In our findings, between the XT1 and XT2 data, there were at most 2 (3.4%) reclassifications across the three model variants (Figure 4). Of these changes, all individuals moved up a risk category, indicating that, in general, all three variations of the μFRAC model tend to marginally overpredict the fracture risk. This means that using the model with XT2 data is not likely to result in an individual who is at risk of fracture based on μFRAC from being overlooked. The added value of HR-pQCT in fracture risk prediction [26] is further reinforced by the robustness of the fracture risk prediction across scanner generations.

We were encouraged that the performance of both the phenotyping and μFRAC models was highly reproducible on both XT1 and XT2 data. The categorical phenotype classification on the XT2 precision cohort was lower than the XT1 cohort; however, the correlation between the membership values was very high for both data sets. This is due to individuals in the XT2 precision cohort equally belonging to more than one phenotype, meaning minor changes in their phenotype memberships leads to a change in categorical classification. Despite this, all changes in phenotype classification tended away from the healthy phenotype. The high level of reproducibility across the models indicates high model stability on both XT1 and XT2 data, meaning regardless of the scan generation used the models perform equally as consistently. Additionally, although individuals were excluded from the data set as a result of a high motion score (>4) when all individuals were included in the analysis, no notable difference was seen in the results. We found that individuals with higher motion scores did not have a greater difference in model outputs; this is likely a result of the quality of the majority of input parameters used in these models not being highly affected as a result of motion [27]. This indicates that both the bone phenotyping and μFRAC models are robust to motion artifacts.

The model robustness is reflected by similar precisions for both XT1 and XT2 (Table 1). For the bone phenotyping model, all three of the phenotypes performed equally, indicating that the model is stable and highly reproducible. For the μFRAC variants, the μFRAC" model had a lower RMSSD and LSC value, which suggests that where possible the full μFRAC model is preferred, although the RMSSD and LSC were low for all model variants.

The difference in scan region between XT1 and XT2 did not appear to affect the outcomes of the models. In XT2 scanning, the scan region extends 1.2 mm proximal to XT1, meaning that additional proximal bone is captured [28]. Although we technically could include only the XT2 data that overlapped with the XT1 region, we intentionally used the whole scan region because in practice it is how any future use of XT2 data would be used. This difference in scan region undoubtedly affects HR-pQCT outcomes, for example an increase in total bone mineral density or cortical thickness. However, despite this difference in scan region, both phenotyping and μFRAC models appeared to perform with a high level of accuracy when applied using the XT2 data as input. Because previous studies have shown that the difference in outputs provided by XT1 and XT2 can largely be resolved using cross-calibration [18, 24], we also tested if cross-calibration applied to the XT2 data could improve the accuracy of the phenotyping and μFRAC results using XT2 data. We found marginally increased accuracy (data not shown); however, this additional step and added complexity did not justify its implementation in our opinion. In addition, this could indicate that other slight variations in scan regions (ie fixed offset compared with relative offset, or variations in fixed offset distances) would similarly be expected to have a marginal impact. A recent study in our lab comparing the use of the fixed and relative offset positioning indicates that due to the inherent variability of underlying bone anatomy, it is reasonable to compare studies using different positioning methods [29]. Furthermore, our second-generation data positioned 0.5 mm more proximally than the current guidelines likely has minimal effect on measurement outcomes, and in fact would be equivalent to a 95% overlap in a longitudinal study. Given the inherent variability in the anatomy, we feel that our μFRAC and phenotyping models are robust to scan positioning and therefore are likely broadly applicable for HR-pQCT imaging, although not explicitly tested.

A limitation of this study is that 58 individuals scanned using both systems may not capture the full range of microarchitecture that would be expected in a population. A cohort with paired scans from both generations is rare and we were fortunate that our cohort included individuals from the three phenotyping classifications and a broad range of fracture risks. The distribution of bone parameters seen in the cohorts was comparable (Figure S1, see online supplementary material for a color version of this figure), and the demographic information was similar (Table S1). Second, the bone phenotyping model predicts fuzzy clusters, meaning that one individual can similarly belong to more than one phenotype (if they exhibit characteristics from multiple phenotypes); therefore, small changes to the model input could reasonably lead to a different classification of the categorical phenotype. Finally, this study focuses on the viability of the models on XT2 data and cannot be used to validate the predictive accuracy of the models in XT2. To validate and compare the use of the μFRAC model on XT2 data to other fracture risk prediction models, a large XT2 prospective study cohort is required. Ongoing work is being conducted to validate the μFRAC model in independent population-based cohorts that capture prospective fragility fractures in both males and females (eg [30, 31]).

In conclusion, we demonstrated that bone phenotyping and μFRAC models trained using XT1 cohorts generalize well to XT2 data. The predicted outputs from the models were consistent between both scanner generation, and any changes in outcome from XT2 data tended to be more conservative. Therefore, where a suitable XT2 cohort is not available to retrain a model, the original XT1 trained model can be used with a reasonable level of confidence.

## Supplementary Material

Supplementary_Material_zjae039

## Data Availability

Processed data will be made available upon reasonable request.
