# Supplementary material for: Transferability of bone phenotyping and fracture risk assessment by μFRAC from first-generation high-resolution peripheral quantitative computed tomography to second-generation scan data
Source: J Bone Miner Res. 2024 Mar 4;39(5):571–9. doi: 10.1093/jbmr/zjae039 (PMC11262140; doi:10.1093/jbmr/zjae039)
Supplement: Supplementary_Material_zjae039 [file Supplementary_Material_zjae039.docx]

**Supplemental Table 1:** Descriptive and summary statistics of the cohorts included in this study compared to BoMIC [1].

|  | **Parameter** | **BoMIC** | **XT1 Generalizability Cohort** | **XT2 Generalizability Cohort** | **XT1 Reproducibility Cohort** | **XT2**  **Reproducibility Cohort** |
| --- | --- | --- | --- | --- | --- | --- |
| **Radius** | TtBMD (mg HA/cm^3^) | 294.7 ± 67.5 | 299.2 ± 58.0 | 306.9 ± 63.0 | 295.7 + 58.0 | 315.8 ± 67.0 |
|  | CtBMD (mg HA/cm^3^) | 832.7 ± 77.2 | 922.3 ± 49.0 | 893.3 ± 54.8 | 865.6 ± 70.3 | 887.3 ± 51.4 |
|  | TbBMD (mg HA/cm^3^) | 149.9 ± 41.9 | 159.6 ± 41.4 | 142.5 ± 42.2 | 153.3 ± 37.7 | 153.6 ± 42.4 |
|  | TtAr (mm^2^) | 290.3 ± 75.9 | 301.0 ± 59.1 | 252.1 ± 52.9 | 294.4 ± 56.1 | 306.9 ± 73.5 |
|  | CtAr(mm^2^) | 51.0 ± 16.9 | 55.8 ± 15.5 | 56.1 ± 14.4 | 59.4 ± 13.4 | 67.8 ± 17.1 |
|  | TbAr (mm^2^) | 231.6 ± 68.9 | 240.5 ± 50.1 | 199.5 ± 45.8 | 238.9 ± 50.0 | 243.1 ± 67.2 |
|  | TbN (mm^-1^) | 1.80 ± 0.37 | 2.0 ± 0.3 | 1.3 ± 0.2 | 1.9 ± 0.3 | 1.4 ± 0.2 |
|  | SdTbN (mm) | 0.25 ± 0.18 | 0.3 ± 0.1 | 0.3 ± 0.1 | 0.2 ± 0.1 | 0.3 ± 0.1 |
|  | CtTh (mm) | 0.70 ± 0.21 | 0.9 ± 0.2 | 1.0 ± 0.2 | 0.9 ± 0.2 | 1.1 ± 0.2 |
|  | FE Failure Load (N) | 2253 ± 701 | 2156.6 ± 914.2 | 2053.3 ± 658.3 | 2454.2 ± 645.0 | 3313.2 ± 1097.1 |
| **Tibia** | TtBMD (mg HA/cm^3^) | 269.1 ± 56.6 | 282.9 ± 53.2 | 278.9 ± 54.0 | 281.3 ± 51.0 | 304.3 ± 61.3 |
|  | CtBMD (mg HA/cm^3^) | 810.8 ± 72.7 | 862.8 ± 56.5 | 848.3 ± 61.4 | 829.5 ± 79.4 | 855.0 ± 56.1 |
|  | TbBMD (mg HA/cm^3^) | 160.3 ± 39.0 | 176.2 ± 39.5 | 159.8 ± 38.3 | 173.5 ± 32.8 | 175.4 ± 37.8 |
|  | TtAr (mm^2^) | 728.7 ± 147.3 | 740.3 ± 137.9 | 664.3 ± 120.8 | 741.2 ± 128.5 | 755.4 ± 141.3 |
|  | CtAr (mm^2^) | 107.5 ± 34.4 | 112.5 ± 25.2 | 114.5 ± 23.6 | 120.4 ± 27.0 | 142.4 ± 33.4 |
|  | TbAr (mm^2^) | 611.3 ± 139.9 | 620.8 ± 132.4 | 555.0 ± 116.9 | 626.7 ± 122.6 | 618.5 ± 134.8 |
|  | TbN (mm^-1^) | 1.8 ± 0.4 | 1.9 ± 0.3 | 1.3 ± 0.2 | 1.8 ± 0.4 | 1.3 ± 0.2 |
|  | SdTbN (mm) | 0.3 ± 0.2 | 0.3 ± 0.1 | 0.3 ± 0.1 | 0.2 ± 0.1 | 0.3 ± 0.1 |
|  | CtTh (mm) | 1.0 ± 0.3 | 1.2 ± 0.2 | 1.4 ± 0.3 | 1.2 ± 0.2 | 1.6 ± 0.4 |
|  | FE Failure Load (N) | 5831 ± 1536 | 6556.6 ± 2095.8 | 5661.7 ± 1418.4 | 6508.7 ± 1256.5 | 9114.9 ± 2460.7 |
| **DXA** | FN aBMD (T-score) | -1.2 ± 1.0 | 0.4 ± 1.1 | | -0.4 ± 1.1 | -0.7 ± 0.9 |
| **Demographics** | Previous Adult Fracture (% Fractured) | 1325 (22.6 %) | 25 (43.1 %) | | 15 (40.5%) | 12 (16.2 %) |
|  | Sex (% Female) | 4165 (70.9 %) | 41 (70.7 %) | | 28 (75.7 %) | 35 (47.3%) |
|  | Age (years) | 68.0 ± 8.6 | 66.4 ± 4.0 | | 58.1 ± 8.6 | 66.2 ± 4.5 |
|  | Height (cm) | 163.3 ± 8.6 | 157.9 ± 8.0 | | 153.2 ± 33.4 | 169.0 ± 8.2 |
|  | Weight (kg) | 70.9 ± 15.1 | 74.2 ± 14.3 | | 88.3 ± 38.6 | 80.0 ± 14.3 |

**Supplemental Figure 1:** Parameter distribution histograms of the cohorts included in this study compared to BoMIC [1].


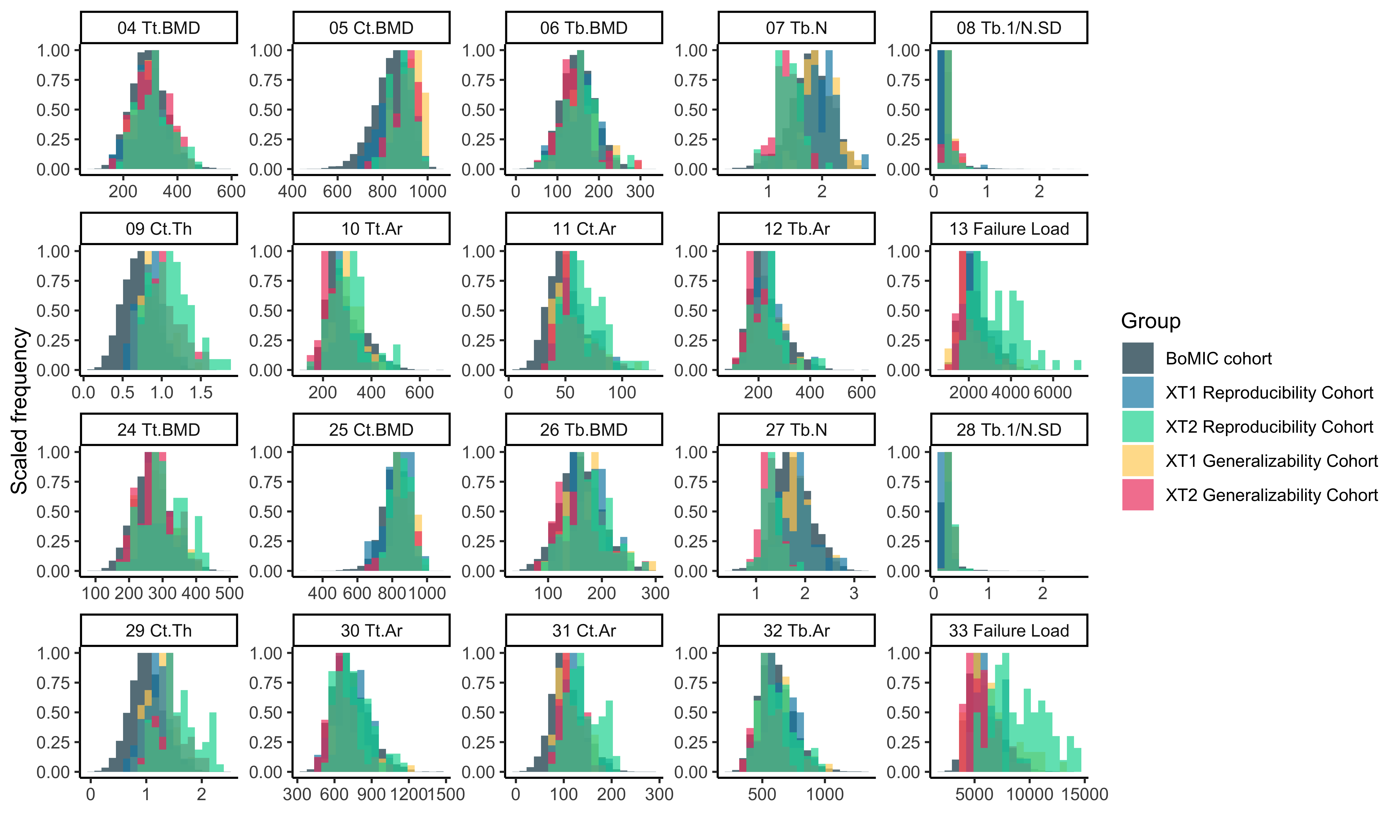


[1] E. J. Samelson *et al.*, "Cortical and trabecular bone microarchitecture as an independent predictor of incident fracture risk in older women and men in the Bone Microarchitecture International Consortium (BoMIC): a prospective study," *The lancet Diabetes & endocrinology,* vol. 7, no. 1, pp. 34-43, 2019.
